# Cancer as a death sentence: developing an initial program theory for an IVR intervention

**DOI:** 10.1093/heapro/daac070

**Published:** 2022-08-01

**Authors:** Onaedo Ilozumba, Johnblack Kabukye, Nicolet de Keizer, Ronald Cornet, Jacqueline E W Broerse

**Affiliations:** Applied Health Sciences, University of Birmingham, Birmingham, UK; Uganda Cancer Institute, Kampala, Uganda; Department of Medical Informatics, Amsterdam Public Health research institute, Amsterdam UMC—Location AMC, Amsterdam, The Netherlands; Uganda Cancer Institute, Kampala, Uganda; Uganda Cancer Institute, Kampala, Uganda; Faculty of Science, Vrije Universiteit, Amsterdam, The Netherlands

**Keywords:** mHealth, cancer, health promotion, low resource settings, participatory research

## Abstract

To address current trends in poor health-seeking behaviour and late cancer diagnosis in many low- and middle-income countries, like Uganda, it is important to explore innovative awareness building interventions. One possible intervention is a common digital format, an interactive voice response (IVR) system, which is suitable for individuals with low technological and reading literacy. It is increasingly acknowledged that developing digital interventions requires co-creation with relevant stakeholders and explication of program developers’ assumptions, to make them effective, sustainable, and scalable. To this end, we sought to develop an initial program theory for a co-created IVR system for cancer awareness in Uganda. Utilising principles of the realist approach, a qualitative exploratory study was conducted through seven focus group discussions (FGDs) with people living with cancer (PLWC), health workers, and policy makers. Thematic analysis of the transcripts resulted in the emergence of four major themes. Through all themes the most consistent finding was that myths, misconceptions, and misinformation about cancer were related to every aspect of the cancer journey and influenced the experiences and lives of PLWC and their caregivers. Participants were positive about the potential of an IVR system but also had reservations about the design and reach of the system. The resulting initial program theory proposes that a context-specific IVR system has the potential to improve awareness on cancer, provided attention is given to aspects such as language, message framing, and accuracy.

## INTRODUCTION

The burden of cancer is growing globally, including in countries like Uganda where survival rates remain suboptimal (Bray *et al.*, 2018) and low awareness, stigma, and misconceptions remain widespread (Mwaka *et al.*, 2014; Meacham *et al.*, 2016; Ndejjo *et al.*, 2017a). Research suggests that lack of awareness and misinformation about cancer, including fatalistic perspectives and negative cancer beliefs, are linked to poor health-seeking behaviour and poor cancer survival outcomes (Von Wagner *et al.*, 2011; Pedersen *et al.*, 2018). Addressing these gaps is made more challenging by the range of possible cancer symptoms, diagnoses, and treatment options. Additionally, the general population and PLWC (and their caregivers) have different information needs.

As a result of this complexity, cancer awareness programs in Uganda are often cancer specific (e.g. focused on cervical cancer), focused on screening or early detection, and delivered via multiple interventions, such as radio programs, outreach sessions by health professional, community health workers as educators, and use of printed materials (Nakandi *et al.*, 2013; Mukama *et al.*, 2017; Scheel *et al.*, 2017). However, these programs also face multiple challenges, including barriers related to the shortage of human resources for health and health funding, making it imperative to innovate delivery methods for cancer information (Morhason-Bello *et al.*, 2013; Mwaka *et al.*, 2013; Nakaganda *et al.*, 2021). In the last decade, the ubiquity of mobile phones has led to increased attention of digital solutions, such as mobile health (mHealth), as potentially effective, and low-cost ways to address the information gaps within the general society as well as cancer patients and caregivers (Dickinson *et al.*, 2014; Charalambous 2019).

mHealth involves the use of mobile technology to provide healthcare services and could include voice, text messaging (short message service, SMS), and multimedia message services and applications. One form of mHealth is interactive voice response (IVR), a technology that allows clients to receive pre-recorded audio information over the phone by interacting with a computer-based telephone system via touch tones or voice commands (Lee *et al.*, 2003; Kraft and Androwich 2012). IVR technology utilizes the mobile phone infrastructure to cost-effectively deliver health information to a large audience, while reducing the pressure on the health workers force and the need for patients to travel long distances to health facilities to get health education from health workers. There is some evidence supporting the use of IVR systems on a range of health conditions. Among young adults living with HIV in Uganda, IVR was found to be acceptable and promote anti-retroviral drug adherence and increase health knowledge (Twimukye *et al.*, 2021). Another study in Uganda and Nigeria on fistula barriers reported positive impressions of the IVR, especially the ability to preserve anonymity in seeking information and referral for fistula symptoms. (Tripathi *et al.*, 2020). Both studies reported challenges including limited mobile phone ownership, software failure, and poor cellular network connectivity. From similar contexts such as Ghana there is evidence that IVR was deemed acceptable to users (clinicians and parents of sick children) (Brinkel *et al.*, 2017; Franke *et al.*, 2018). Reviews on IVRs have also found effects on medication adherence, physical activity, (Tsoli *et al.*, 2018) and cardio-metabolic medications (Kassavou and Sutton, 2018). The reviews also emphasized behavioural change messages about ‘health consequences” and messages that are “tailored”, i.e. sending different messages to different participants/patients as success pre-requisites. However, there is currently no evidence on IVR for cancer awareness in the Uganda context.

Despite the promise of IVR and other mHealth interventions, reported program outcomes are often inconsistent. It has been suggested that this can be related to program developers failing to make explicit the assumptions related to program activities and outcomes (Ilozumba *et al.*, 2018). mHealth interventions are multi-layered and complex, regardless of the assumed simplicity of the technology itself. The interventions must be acceptable and utilized by the intended end-users and these are often influenced by multiple factors, including characteristics of the users, the technology as well as other contextual factors, which could influence the performance of desired behaviour. One way to improve the outcomes mHealth interventions is to adopt a nuanced approach towards understanding programs. In this study, we propose the utilisation of the Realist Evaluation (RE) approach to address complexity.

Realist evaluation seeks to understand ‘what works, for whom, under what circumstances, and how’ (Pawson and Tilley, 1997). Realist evaluation is generative as it operates under the assumptions that mechanisms are only activated with specific suitable contexts. The first stage of a RE is the development of a program theory, which generally seeks to move beyond a more simplistic logic framework that attempts to explain how outcomes are achieved. Rather, program theories in RE incorporate a theory of change, and present multiple hypotheses that make explicit how outcomes may be achieved, via what mechanisms and in which contexts. These hypotheses are often referred to as context-mechanism-outcome configurations (CMOs), which could result in a program theory. Program theories can be conceptualized more specifically or at higher levels of abstraction. In this study, we adopt a narrow definition of program theory which hypothesizes the pathway by which a specific intervention leads to a goal (Davidoff *et al.*, 2015). An initial program theory for RE ideally is developed at the time of the design of a new intervention and refined through the intervention implementation and evaluation.

A well-developed program theory must involve input of multiple stakeholders to develop a holistic understanding. This is incorporated in the principles of co-creation, which purposes that knowledge should be created not only by academics but also by other stakeholders including the intended users of a service (Greenhalgh *et al.*, 2016). Co-creation holds promise for research in which there is the intention to innovate solutions that solve human experience problems. In this study, we focus on co-creation with potential end-users (PLWC and care-givers) and stakeholders (Leask *et al.*, 2019).

In this study, we aimed to develop an initial program theory for a co-created IVR system for cancer awareness in Uganda by conducting a qualitative exploratory study. This is critical, as our project to utilize IVR for cancer information needs in Uganda, does not, to the best of our knowledge, have any precedent. The development of an initial program theory also allows for an exploration of the existing CMOs within which the intervention will be implemented. Therefore, the initial program theory development was guided by the question ‘Is the use of IVR technology for cancer information provision acceptable and feasible within this context and for relevant stakeholders?’

## MATERIALS AND METHODS

To develop an initial program theory, it is essential to understand (i) the general population’s awareness and understanding of cancer, (ii) the lived experiences of PLWC and their caregivers, and (iii) perceptions and attitudes of stakeholders about the IVR system. We employed theoretical understanding gained from the unified theory of acceptance and use of technology (UTAUT) and the health belief model (HBM). Key informant interviews (KIIs) and focus group discussions were conducted with multiple stakeholders in July and August 2019 at the Uganda Cancer Institute, Kampala.

### Contextual background—Uganda

Cancer care in Uganda is provided primarily by the Uganda Cancer Institute in Kampala, the only publicly funded cancer institute in Uganda. This means that most of the population lives at some distance from affordable and good care. IVR is commonplace in customer relations in many businesses (telephone, internet, and TV providers), whereas mobile health solutions based on voice telephony (e.g. call *centres*) are also common, particularly for HIV and reproductive health advice (Kamulegeya *et al.*, 2020, Byonanebye *et al.*, 2021). Furthermore, the SMS platform mTrac has been used by the Uganda Ministry of Health for collection of healthcare indicators and to engage health workers (Rassi *et al.*, 2018). However, IVR technology or mobile health solutions in general have not been used before for provision of cancer information in Uganda, nor in any other country in Sub-Sahara Africa.

### Theoretical background—UTAUT and HBM

The success of an mHealth intervention depends on a complex interplay of factors, beginning with the acceptance of the proposed technology. There is significant evidence to suggest the acceptability of mHealth interventions, including maternal and neonatal health, diabetes care, and medication adherence. The Unified Theory of Acceptance and Use of Technology (UTAUT) was developed initially by Vankatesh *et al.* (Venkatesh *et al.*, 2003) as a response to the growing need for a model which addressed understanding of technology acceptance as the first step to intervention effectiveness (Venkatesh *et al.*, 2003). UTAUT was developed by empirically testing and comparing eight models (including the theory of planned behaviour, innovation of diffusion, and technology acceptance model) that address *how* and *why* individuals adopt new technology. The result was a unified model that identified four constructs as most likely to be significant determinants of technology acceptance: (i) *performance expectancy* (an individual’s belief that a system can help), (ii) *effort expectancy* (degree of ease associated with the use of the system), (iii) *social influence* (expectation of others that an individual should use a system) and (iv) *facilitating conditions* (belief in the existence of organisation and infrastructural support). Moderators of the behaviour were gender, age, voluntariness, and experience.

Additionally, while UTAUT captures issues related to the technology, we believed it was also important to include an understanding of the individual’s perceptions of illness itself. Therefore, we also incorporated the health belief model (HBM) to understand perceptions of cancer among our stakeholders. The model consists of four key constructs: (i) *perceived susceptibility* (belief about likelihood of getting a disease), (ii) *perceived severity* (belief about the seriousness of a disease), (iii) *perceived benefits* (of changing a behaviour), and (iv) *perceived barriers* (impediments to undertaking recommended behaviours) (Champion and Skinner 2008). *Self-efficacy* (confidence in one’s ability to act) is another important construct added to revised versions of the model.

### Data collection

Qualitative data collection was conducted in two phases: (i) Exploration and vignette development, and (ii) Needs assessment and program theory development. Given the exploratory nature of this study and the objective of the RE, i.e. nuanced understanding of program theory (contextual issues, mechanisms, and outcomes), qualitative methods were deemed most suitable.

#### Phase 1: Exploration and vignette development

To obtain rich data about a potentially sensitive topic, we adopted an approach of using vignettes as data collection tools. Vignettes are usually short stories consisting of text and/or images which participants respond to (Hughes and Huby 2002). Vignettes that recreate real-life situations for participants interested in a topic have been found to be a useful tool for maintaining participant interest (Hughes and Huby 2004). To develop the study vignettes KIIs with three healthcare professionals and a group interview with three PLWC were organized. Interview guides for the KIIs were based on the concepts of UTAUT and HBM. The interviews were conducted by authors OI and JKK and lasted 30–60 min. The transcripts from these interviews were analysed and four vignettes were subsequently developed and reflected the most discussed experiences and situations that in literature review and interviews.

Three vignettes presented as a hypothetical person: a man (Nsereko) with prostate cancer, one of the most common forms of cancer. The first three vignettes followed Nsereko’s cancer journey from the moment he reported symptoms to his first wife (Saanyu) until his diagnosis and treatment at the UCI. Given that the main character is male, we thought it important to also include a female character living with cancer. This would provide an opportunity to elicit any gender or cancer specific findings. Thus, we developed a fourth vignette which introduced a woman living with breast cancer (Supplementary File 1) . Cervical cancer was excluded as exploratory interviews revealed that increasing knowledge on the human papillomavirus (HPV) as a sexually transmittable virus was leading to stigmatization of cervical cancer as a sexual cancer.

#### Phase 2: Needs assessment and program theory development

Recruitment was undertaken for focus group discussions (FGDs). Purposive sampling was utilized to recruit FGD participants; this involved the careful selection of individuals who were able to provide rich information about the cancer needs of patients and healthcare providers at UCI as well as the broader context of Uganda. For this study, such individuals belonged to one of three stakeholder groups: PLWC (men and women) in different phases of the cancer journey, health workers of different cadres, and professional roles (doctors, oncologists, nurses, health educators, counsellors) and policy makers (e.g. from the Ministry of Health, Uganda Communications Commission, and other regulatory bodies). Criteria for each group can be found in Table 1.

**Table 1:** Overview of sampling criteria and characteristics of study participants

Sampling criteria of participants	
Group	Criteria
PLWC caregivers	1. Be at one of the four phases of the cancer journey (detection, diagnosis, treatment, survivorship) 2. Represent one of the most common cancer types (cervical cancer, prostate cancer, breast cancer, Kaposi’s sarcoma, Burkitt’s lymphoma, lung cancer, skin cancer, cancer of the bone, cancer of the eye, cancer of the colon, and cancer of the blood.) 3. Currently receiving treatment at UCI or have received treatment there in the last 12 months 4. Or be a caregiver of a person who fits the above characteristics 5. Able to communicate in English or Luganda Inclusion: ((1 AND 2 AND 3) OR 4) AND 5
Health professionals	1. Staff of UCI or Palliative care team from Mulago cancer hospital in Kampala 2. Directly involved in patient care 3. Complement represented cadres of health professionals Able to communicate in English or Luganda
Policy makers	Work within governmental, non-governmental, or patient organizations with a focus on cancer care

Overview of study participants characteristics	
Characteristics	*N* (%)
Interviews 2 Health professionals and 1 Patient	3 (4)
FGD participant***s***	
* *PLWC/Caregivers	20 (27)
* *Health professionals	39 (53)
* *Policy makers	11 (15)
Gender	
Females	41 (56)
Males	32 (44)
Age range	21–70
Mean age (SD)	37.1 (9.1)
Education level	
Primary school or none	19 (26)
Secondary school	10 (14)
College diploma	16 (22)
Bachelor’s degree	21 (29)
Master’s degree or higher	7 (10)
Total	*N* = 73

Five FGDs were conducted in English. Two FGDs with patients and caregivers were conducted in Luganda to ensure the participation of stakeholders who were less comfortable communicating in English. After seven FGDs as data collection was reached and no further interviews were conducted (Saunders *et al.*, 2018). Additionally, within all FGDs participants were encouraged to express themselves in specific local language phrases or words if that was easier for them, these were then translated at the transcription stage. The FGDs were facilitated by OI, JKK, and trained research assistants, they lasted 90–120 min. A total of 73 participants took part in seven FGDs over 2 weeks.

The average age of PLWC and caregivers was 41.5 years, and about half of PLWC and caregivers was married (56%). While 60% of all participants had higher than secondary school education, this included health care professionals and policy makers. Of the PLWC and their caregivers 38% had some primary education, 41% had secondary education, and 21% had some post-secondary school education.

### Data analysis

All transcripts were translated to English and translated verbatim. Translation and transcription were performed by trained research assistants who were native speakers of the required language. All transcripts were analysed using MAXQDA. A thematic analysis approach was applied, beginning with open coding of two transcripts by OI and JKK. Both independent codebooks were discussed, and final codes agreed upon and defined. The codebook that emerged from this process was utilized to analyse all transcripts. All transcripts were then coded by OI and JKK with emergent codes added to the transcript as they arose. After coding, OI and JKK revised the codebook based on emergent codes and discussed axial codes and themes occurring in the data. A detailed overview of this codebook is presented in our previous publication on the implementation of the IVR system (Kabukye *et al.*, 2021). In this manuscript we report on the development of CMOs and ultimately the initial program theory.

### Ethics

The study was approved by the UCI research ethics committee (UCIREC# 08-2019) and was registered by the Uganda National Council for Science and Technology (UNCST# HS418ES). All participants provided written informed consent before taking part in interviews, FGDs. Participants received reimbursement for their time and inconvenience in accordance with the rates stipulated by the UCI research ethics committee (approximately US $13).

## RESULTS

Four main related themes emerged, namely: (i) health-seeking delays, (ii) barriers in diagnosis and treatment trajectory, (iii) social support, and (iv) acceptability and usability of IVR System. The initial program theory CMO configurations were developed during analysis and are presented at the end of the results section.

### Health-seeking delays

The main three factors related to health-seeking delays were (i) myths and misconceptions, (ii) perceived deficiencies in the health system, and (iii) lack of accurate information.

In all FGDs, participants discussed the *myths and misconceptions* related to cancer in their communities as an important barrier to health-seeking behaviours. Topics of discussion were the intersection between the belief in unexplained symptoms because of witchcraft and the faith placed in traditional medicine practitioners. Many individuals, when confronted with an unknown ailment that is not easily categorized, believed that the ailment was of supernatural origin and would consult multiple traditional medicine practitioners before seeking care in the hospital. This pattern held through for almost all PLWC and caregivers in the FGDs.

*In most cases, when people get such cancer-related problems, they always think it is witchcraft. That leads to late-coming to hospital. Some people will spend money at Mutulaakungo (a witch who dares to sit on a leopard), they offer goats and cocks. The Mutulaakungos can never admit that they will not manage a problem.*

*(P2_male_FGD)*

Christian faith was reported to have mixed effects on cancer-related health-seeking outcomes. For some patients Christianity meant believing that cancer symptoms were of supernatural origin and required prayers and supernatural interventions. However, for some participants, this belief in witchcraft as the cause of cancer was over-ridden by Christian faith, this resulted in seeking care from the hospital and not traditional medical practitioners:

*Sometimes, it depends on your faith. I was a born-again Christian, an active church member. I believed that God would heal me. Because I had faith, I did not believe in witchcraft. I first went to a medical centre, when I did not improve, I decided to come to Mulago [National Referral Hospital]. People were telling me that my problem looked similar to another man’s problem who was known to have been bewitched, that I should go the same witch doctor who had healed him. And the problem is; in most cases when people learn that you have cancer, they think you are dying any time because of the belief that there is no cure for cancer.*

(P2_male_FGD)

In the discussions all FGDs participants agreed that many individuals might only have heard of cancer as a distant disease which results in death, because it was generally thought that cancer cannot be cured. Hence, cancer is neglected, and health-seeking is delayed.

*They called me one year back a walking dead…So based on such kind of fears Nsereko might feel [if I believe] I have cancer. I’m going to die soon I am going to be dead the same idea it is going to be very hard for Nsereko to believe that he has cancer. Until maybe if the information is provided with him about cancer. Or these systems that you are coming up with if he gets information that there is a cancer called prostate cancer which has these effects, maybe I go to screen. But to believe at the first point that you have cancer is not an easy thing.*

*(P4_male_FGD)*

In addition to the myths and misconceptions, participants perceived that the decision to consult traditional medicine practitioners was also driven by *perceived deficiencies in the health system*. The traditional medicine practitioners were often located closer to the community, and they were easier for individuals to access as opposed to medical health centres, which were generally at a long distance, expensive, and confusing to navigate.

*One other thing that causes delays is lack of equipment/gadgets in the lower health centres. The doctors in those units get challenges due to lack of “things” to use. They end up referring people to Mulago [National Referral Hospital]. What happens, one will have to first go home to look for money because you cannot dare to come to Mulago without money. In that process, you keep getting advice about other options. You know, the traditional healers do not take a lot of money.*

*(P3_male_FGD)*

Most participants also mentioned the *lack of accurate information*. Reflecting on the sources of accurate cancer information, multiple sources were discussed including mass media (radio and television), health centres, and churches. Regardless of the information modality, the majority expressed a belief that women were often in possession of more information about cancer than men. This was attributable to five key factors: (i) women’s reproductive health needs and roles as key caregivers in the family led to greater contact with health professionals, (ii) in their role as major caregivers the possibility that a woman could have been involved in caring responsibilities for a friend, family, or community member with cancer was higher, (iii) women were more likely to attend church services and programs and thus receive information there, (iv) women were described as being more social and likely to share life experiences in the communities and with others, and (v) an increase in activities geared at providing breast and cervical cancer screening, meaning that women have additional exposure to cancer information.

*Many factors contribute to women being more of being or visiting facilities than men. Ah, they may not specifically be the ones that are sick, but if you do research you find that they are the ones involved in giving care. So, it could be her sister, it could be her mother, it could be her child. So, most times, rarely do you find, the majority of men in health facilities. So, given the fact that it is women that visit the health facilities, they are more exposed to getting information than the men. And on the other hand, men are more on, yah, they are looking for businesses, they want to get money, by the time the man goes to hospital … [laughs] he is really, really, really sick that he has no option but to go to hospital… [laughs]*

*(P7_Health worker_FGD)*

It is important to note that this perception of women’s knowledge does not consider whether the information received was accurate or if receiving more information was indicative of receiving sufficient information. Additionally, while access to accurate information can improve knowledge, there are many other variables that result in higher knowledge and health-seeking behaviours (e.g. ability to understand the information).

Participants also discussed other PLWC and health institutions as sources of information. However, in all responses there was a component of luck in receiving information, participants had to meet the right patient, or report at the right health centre, be at church or listening to the radio at the right moment to receive the information. Participants were unaware of any sources via which PLWC, and caregivers could actively seek relevant information at their own convenience.

### Barriers in diagnosis and treatment trajectory

Participants discussed their experiences during their diagnosis and treatment, including uncertainty of the credibility of information on cancer, issues they faced navigating the cancer referral process and the UCI itself, and experiences with and concerns about health services.

Once participants received a referral to the cancer hospital Mulago in Kampala they still faced multiple challenges. Key amongst these was an *uncertainty of the credibility of information* received at the location and difficulty knowing what their next steps were. Most participants lived outside of Kampala and faced challenges getting to Kampala, then on arriving at the hospital they had issues navigating the hospital environment. There were often individuals providing contrasting and sometimes factually inaccurate information leading to confusion and anxiety.

*When you are not familiar with Mulago and try to ask people around, you get a lot of discouraging feedback. The moment you show people that you do not know where to start, someone will even ask if you have a million shillings. The moment you say you do not have money; they tell you that you have hit the dead end. Someone will lie to you that they had to spend one million, another one two million. You end up giving money to anybody be it a cleaner.*

*(P1_female_FGD)*

The PLWC and caregivers discussed *difficulties with navigating the cancer referral process and the UCI*. They expressed a shared belief that Nsereko on arriving at the hospital and receiving his diagnosis would have minimal knowledge of what to expect from the cancer diagnosis and treatment process. One shared experience was the very unusual nature of cancer diagnosis and treatment. Most participants arrived at Mulago expecting to receive a diagnosis immediately along with a clear treatment path to a cure. This is, despite the discussions about the prevailing believes that a cancer diagnosis is a death sentence, participants still expressed a hope for a good prognosis.

*I’d wanted to say that because Nsereko doesn’t know much about cancer and he doesn’t know if cancer treatment has stages. Yeah, so I don’t believe he will ask which stage he has. To me the thing he is going to ask the doctor is ‘how long will I take here’? That will be the first question to ask. So he is thinking that may be after getting a cure he is going to go back so he will ask how long am I going to take here with the treatment. May be after asking that question the idea of which treatment should I go on to get cured?*

*(P10_Male_FGD)*

Participants overall expressed positive *experiences with the health professionals* they met during their treatment. Those participants who interacted with paediatric oncology services were particularly complimentary of the treatment and care they received. However, the interactions with the doctors were often fraught with confusion and fears about exactly what the diagnosis meant, imminent death, worries about the financial implications of their treatment as well as other negative effects on their family members such as children. These concerns and limited face-to-face time with health professionals meant that often participants would not be able to process information received and ask follow-up questions at scheduled appointments.

### Social support

The importance of social support in the form of understanding from family and community, financial support, and moral support were consistently highlighted in the interviews and FGDs.

Overall, participants experienced very low *support from family and community*. Given the prevailing perceptions of cancer as a death sentence, the attitudes of family, and community members could range from avoidant to outright abandonment. There seemed to be no distinction between family and community as many participants shared stories of parents and relatives urging them to accept their inevitable death and refrain from seeking additional care. Participants discussed that one of Nsereko’s most persistent fears would be of abandonment by his wives, perhaps for other men. Participants discussed women not wanting to be with a man who could not work or perform sexually. An overarching reason for the general experience of abandonment was the financial burden for caring for a person expected to die.

*… the thoughts of the community and family members towards Mr Nsereko, they may have abandoned him like I have seen in the hospitals here, so many people have been abandoned because they have cancer…They have a feeling that cancer cannot be cured, then the next thing is that now if it can [be] cured for how much money? Depending on how the family is, maybe they are these poor people they can’t even raise even 200,000 within a month so they are like how will we manage to buy a drug of 800,000 for around 6 cycles so they will just say ‘aaahaa let him die they just wait for the day they are dead we shall pick the dead body’.”* (P2_Female_FGD)

The introduction of a new character in the Vignette,—Nabirye, a city professional working woman undergoing treatment for breast cancer—led to a discussion of *social support from work and work circles*. The majority of participants in the FGDs expected that, since Nabirye was a cosmopolitan, she would most likely discuss her condition with friends, co-workers, and even her employers. As one participant put it:

*…now as she was in the working class I think she could get sick leave from her workplace and get her treatment after that when she is treated or she gets well she goes back and they resume her work that is what I think.* (P7_Female_FGD)

However, while participants who were not employed themselves in “working class” jobs had the perspective of support, those individuals who were employed shared a different take. From their perspective similar lack of knowledge about cancer existed even in the cities and corporate institutions. They discussed stigmatisation after mastectomy, fear of contagion from colleagues, loss of accrued savings, and ultimately job dismissal.

(*Looking sad*) *Yes, I think that Nabirye, … she will face a lot of challenges among others she may lose the job, (Why?) because the bank cannot do without a teller so they had to recruit somebody else. Other people’s business cannot fail because of you who is sick. Like some of us, we lost jobs because they cannot wait [for us to complete our treatments] so that is one challenge.* (P3_Male_FGD)

Unfortunately, the experienced low support from family, community and work was compounded by mixed experiences of support among other PLWC and caregivers at the hospitals. However, some participants experienced uplifting experiences of meeting survivors or PLWC undergoing treatments who shared accurate information and support.

### Use of IVR system

To understand the potential IVR system for cancer information, participants engaged in discussions about the opportunities and pitfalls of such systems. The main topics which emerged were related to usability, utility, and acceptability.

Although the IVR system was explained during the informed consent procedures, in some FGDs, participants required additional explanations of what an IVR system entails. During the conversation, participants had mixed views on the *usability* of such a system. Some concerns expressed by participants were related to the level of technological literacy of intended users, particularly those in rural areas, the access to mobile phones, and availability of electricity to charge phones. Healthcare workers were less likely to express such concerns and believed that the system could help provide information on cancer, particularly to the public.

Despite concerns about usability, most participants expressed understanding of the *utility* of having a system which could be assessed whenever the user desired.

*I think that system will help people a lot because at least in every household there’s a phone so it will reduce ignorance about cancer. It will help patients to know when to come and if the medicines are in or out of stock because sometimes, we come and find that the drugs are out of stock. So, in the process we waste money. It will help to reduce the fears people have about Mulago because the patients and their families will be able to know that tests and drugs are free in Mulago. I think it should also have a provision of speaking to the doctor because even for customer care in the service sector, there is a provision of speaking to a person if you want.*

*(P1_male_FGD)*

Participants discussed issues related to the *acceptability* of messages One key factor that was discussed in all FGDs was the importance of language in ensuring that the message was comprehensible to the people who were deemed as needing it the most. The variety of languages spoken in Uganda was a major point of concern for the participants, as they wondered if one application could deliver the message in all the required languages. They also felt that the messages delivered should present facts that addressed the most common misconceptions about cancer as well as provide general details about the common cancer diagnosis, treatment, and survivorship.

*Yes. The messages to be put in that system need to be assuring. For example, there is a myth that once you are found to have cancer, Mulago becomes your second home, you will never be discharged. The messages should be positive to eliminate such falsehoods. Design the messages in a way to assure the listener [about] the expected period to realize cure if one follows the doctor’s advice, complete their treatment, or ideas of the number of visits to make. Otherwise, it is widely believed that cancer does not heal.*

*(P6_male_FGD)*

Additionally, participants expressed the importance of an effective marketing campaign to inform communities about the program. It was clear that the existence of the system, beneficial as it might would accomplish little if the intended users were not made aware of it. They gave multiple strategies for this sensitisation exercise, including the utilization of television and radio programs, posters at public centres including health care institutions.

### Initial program theory

#### CMO 1: Addressing myths and misconceptions

An IVR system that addresses existing myths and beliefs (C) offers PLWCs and community members with factual and easy to understand information about cancer aetiology, treatment options, and survival probability (M). System users will become more aware of the misconceptions about cancer and have increased knowledge/awareness (O). Additionally providing tailored cancer information via an IVR system empowers PLWC and caregivers (C) by providing accurate information on demand (M), leading to increased cancer awareness (O).

#### CMO 2: Expectation management

An IVR system that provides contextually accurate information on the process of diagnosis and treatment (C) will provide users with an overview of possible procedures, effects, and outcomes (M). Users of the system have an increased awareness, and potentially understanding, of the treatment procedure, increased trust in the healthcare system and be less susceptible to misinformation (O).

#### CMO 3: Demand-based information provision

An IVR system addresses the limitations in the current information supply systems which are often based on participants’ interactions with health services or consumption of mass media (C), by providing a demand information channel, which can be accessed at the user’s convenience (M). This will allow participants’ access information as often as needed and when needed leading to an increase in awareness of the cancer trajectory (O).

#### CMO 4: Design of the intervention

An IVR system which makes available free factual information accessible on basic mobile phones and delivered in local languages and accents and provides options to ask specific questions (C) will be relatable and accessible to the general public (M). This provision of an information source could limit the prevalence of myths and misconceptions about cancer (O).

## DISCUSSION

This exploratory qualitative study sought to understand the acceptability of an IVR system for cancer information awareness and develop an initial program theory to guide the implementation and evaluation of the IVR system. Our findings suggest that current cancer awareness remains low in Uganda with myths and misconceptions related to every stage of the cancer care trajectory.

One important study finding is the range in awareness and knowledge of multiple cancers in Uganda with an emphasis on only a couple of cancers. Participants in our study discussed that women were more likely to have increased awareness about cancers, particularly breast and cervical, for a number of reasons including an increased interaction with health professionals (Sach and Whynes, 2009). This trend in Uganda is also visible in the number of recent studies from Uganda that have focused on breast and cervical cancer. Although cervical cancer is the most prevalent cancer in Uganda (20%), breast, Kaposi sarcoma, Prostate, and Non-Hodgkin lymphoma all have similar prevalence rates at approximately 7% (Global Cancer Observatory, 2021). Yet, these cancers receive less attention in the design of interventions and programs. By exploring participants’ understanding and perceptions of general knowledge of cancer aetiology in their communities, it was clear that while the concept of ‘cancer’ is not unknown in Uganda, myths and misconceptions persist about the disease, its diagnosis and morbidity. The finding of cancer as caused by witchcraft, spiritual origins, or associated with death is similar with findings in other studies (Suh *et al.*, 2012; Ndejjo *et al.*, 2017b; Ngwenya and Huang, 2018; Scheel *et al.*, 2017).

Myths and misconceptions about cancer can be addressed by presenting accurate information through trusted channels. Common sources of cancer information were radio, television, family, and community members, which is similar to findings in Uganda (Mukama *et al.*, 2017). However, all these modes of communication have the same limitation of being available by chance. Participants cannot actively seek out information as or when it is required. This presents a major problem for the public, but particularly for PLWC and their caregivers. While some proportion of these individuals will have access to books and the internet for information, many will not be able to access complex digital or written information formats due to cost and literacy limitations. This is particularly problematic for PLWC and caregivers, who attempt to negotiate cancer diagnosis, treatment, and survivorship with limited information. The lack of information had implications for their communication with healthcare professionals. While the participants did not indicate issues of abuse such as reported in domains such as maternal care (Sacks and Kinney, 2015). However, there is evidence that there is a cost of ineffective communication between doctors and patients. It results in patients feeling dissatisfied with the information, worsens psychosocial contexts and impacts on their decision-making (Thorne *et al.*, 2005). These are all pertinent to the experiences described by our participants, and provides evidence of a gap which can be served by a tool such as our IVR for providing information (Polit and Beck, 2010).

Our findings suggested that participants found the concept of an IVR for cancer awareness acceptable and of potential value. They highlighted possible barriers to utilisation as the availability of mobile phones, the internet, and electricity as well as digital literacy and tailored messages. These findings are consistent with similar research on digital health in sub-Saharan and other low- and middle-income countries which have consistently found that barriers to technology utilization include lack of infrastructure such as electricity, literacy, and language (Khatun *et al.*, 2016; Kruse *et al.*, 2019; Dansharif *et al.*, 2021). However, our results indicated that after addressing these pre-requisites the next hurdle would be ensuring that, how end-users are informed about the intervention. This is a relevant question, particularly for digital interventions, which are rarely scaled-up or sustained beyond the pilot stage (Jahangirian and Taylor, 2015; Luna *et al.*, 2014). Community awareness and uptake of the intervention would be key to be able to evaluate the intervention’s effect on cancer awareness and potentially health behaviours.

We attempt to synthesize all relevant contexts and mechanisms into an initial program theory provides a working framework for the intervention implementation and future evaluations. By making explicit our assumptions about the required context for our intervention, expected mechanisms, and outcomes, we can explore the ultimate intervention outcomes and identify unintended outcomes. The program theory also provides a framework for the evaluation of the implemented IVR evaluation.

This study provides an example of utilising co-creation principles from the initial conceptualisation of a digital technology intervention. While the value of co-creating interventions is known, few digital interventions truly incorporate the end-users into the development of the messages. By including end users and other relevant stakeholders, issues that were unknown to the researchers were raised, including the gendered aspect of health knowledge, lack of demand-based cancer information, and the challenges of marketing the IVR system. However, this strength is also linked to a key limitation of this study—the inclusion of only PLWC and caregivers currently receiving care at the UCI. These individuals are probably not completely representative of PLWCs and caregivers at the UCI or Uganda. They tended to be individuals who had prolonged contact with the health care professionals and some of them were now actively engaged in cancer awareness in their communities. However, for the purpose our qualitative study, a sample representative of the entire population was not essential, rather we strove to recruit participants who could serve as rich sources of information both about their personal experiences but also their communities in general. Additionally, the contextual knowledge of the researchers and ongoing reflections during analysis provided opportunities to question the research findings (Polit and Beck, 2010). 

In conclusion, there is a need to address the significant gaps in awareness and understanding of cancer among PLWC, caregivers of PLWC, and the general population in Uganda. An IVR system which addresses existing myths using local languages, positively framed, and accurate messages could contribute to increasing cancer awareness.

## Supplementary Material

Supplementary material is available at *Health Promotion International* online.

daac070_suppl_Supplementary_File_1
